# Coping, Meaning Making and Resilience Within the Dutch Reformed Pietist Community During the COVID-19 Outbreak: An Exploratory Qualitative Study

**DOI:** 10.1007/s10943-022-01611-8

**Published:** 2022-08-06

**Authors:** Tobias Cinjee, Hanneke Schaap-Jonker

**Affiliations:** 1 Faculty of Social Sciences, Utrecht University, Padualaan 14, 3584CH Utrecht, The Netherlands; 2 Graduate School of Religion and Theology, Vrije Universiteit Amsterdam, Amsterdam, The Netherlands; 3 Clinical Psychology of Religion, Faculty of Religion & Theology, Vrije Universiteit Amsterdam, Amsterdam, The Netherlands; 4 Rector Centre for Research and Innovation, Christian Mental Health Care, Eleos/De Hoop ggz, Hoevelaken, The Netherlands

**Keywords:** COVID-19, Religious coping, Meaning making, Resilience, The Netherlands

## Abstract

In this qualitative study, we examined how community members of the Dutch reformed pietist community coped with the COVID-19 pandemic, and which religious sources of meaning making and resilience they used during this time of crisis. Based on seven in-depth interviews, we found that the representation of God being ‘above all things’ was prominent in times of close encounter with the coronavirus. In actively interpreting the pandemic, community members tended to stay away from concrete eschatological or ecological interpretations. Rather, the general theme of ‘malleability’ was used and linked to notions of calling and punishment. Furthermore, we identified the importance of community and scepticism towards the government as sources of resilience, whereas thinking about the future of the church was a source of fear and concern.

## Introduction and Theory

### Background

The COVID-19 pandemic impacted religious believers worldwide in many domains of their life. They endured uncertainty concerning their health and economic situation and were confronted with death and distress caused by the coronavirus. Those feelings of uncertainty and distress had an effect on religious behaviour; since the beginning of the virus outbreak, a higher prayer intensity was traced (see Bentzen, 2020). Earlier studies have shown that awareness of one’s mortality increased belief in God and beliefs in the efficacy of divine intervention (Norenzayan & Hansen, 2006). This awareness must have been prominent when hospitals were rapidly filling with COVID-19 patients. Meanwhile, religious services were forbidden or restricted.

Yet, in trying to deal with the pandemic, people found sources of hope, resilience and support within their religious communities — even though interpersonal contact had to take a different shape and form. A study on American Orthodox Jews suggested that faith promoted resilience during the COVID-19 outbreak, and that religiosity and trust in God correlated with less stress (Pirutinsky et al., 2020). Research from the USA furthermore showed that greater perceived belongingness to one’s religious community led to diminished perceived impact of COVID-19 as well as reduced psychological distress (Michaels et al., 2022). Also, coping mechanisms which related to ‘hope’ have been examined and were proved to render positive effects on well-being during the pandemic (Counted et al., 2022).

### Religious Coping and Meaning Making

Within psychology of religion, the process of using sources of resilience from a religious tradition and engaging in religious rituals to cope with distress is often referred to as ‘religious coping’ (Pargament et al., 2013). It has been studied in a variety of contexts (Foy et al., 2011; Park & Blake, 2020) and is integrated in models of meaning making where people’s beliefs play an important role (Park & Hale, 2014). Although the concept of coping has often been applied to particular types of distress, related to individual crises (such as depression, individual experiences with disease and death (Norenzayan & Hansen, 2006)), it can also be used to examine how collective crises, e.g. natural disasters like hurricanes, earthquakes, but also the spread of a deadly disease such as COVID-19, impact individuals and their communities (see, e.g. Park & Blake, 2020). Research from Italy and Poland, for instance, showed that religious coping strategies were revitalized or intensified during the pandemic (Boguszewski et al., 2020; Molteni et al., 2021). In the Dutch context, it was, however, found that religiosity (measured by frequency of praying) was largely unaffected right after the pandemic had started (Reeskens et al., 2021).

Religious coping is defined as ‘a search of significance in relation to the sacred’ (Pargament et al., 2005) and, hence, aims at meaning making and understanding (Park & Folkman, 1997). In models of meaning making, a distinction is made between *global meaning* and *situational meaning*. Global meaning refers to the general systems by which people orient themselves (including general theological frameworks and prominent God representations). Situational meaning comes about in the interaction between a person’s global meaning *‘and the circumstances of a particular person-environment transaction’* and, thus, is the meaning ascribed to a specific event or situation (Park & Folkman, 1997, p. 116; Park & Hale, 2014). There may be an incongruence between those two types of meaning.

We may wonder how religious people appraise meaning of the COVID-19 pandemic and how it relates to their general worldview, faith, and perspective on God (e.g. His alleged influence on this world). Community members will act or reason mostly within the boundaries of their established theological frameworks. However, it may be the case that their theological frameworks are extended or are not the main source of situational meaning regarding the COVID-19 crisis. It may even be the case that one’s appraised meaning of the situation differs from the global meaning within the community. One may, for instance, ascribe more meaning to elements which are seen as non-essential in theological theory, such as the importance of community and rituals. It is also likely that the lack of physical meetings and restrictions on church attendance during lockdowns affected people’s perceptions of community, belonging, and congregating (see, e.g. Goyvaerts & Wouda, 2020).

### Religious Coping for Dutch Reformed Pietists

In the current explorative study, we explore how processes of coping and meaning making have been concretized within the Dutch reformed pietist (*bevindelijk gereformeerde*) community,^1^ in the first year of the COVID-19 pandemic. The reformed pietist community in the Netherlands can be found in the ‘Bible Belt’ and forms a well-organized societal group with its own institutions, newspaper, schools and political party (SGP), which are all typical *identity markers* (Klaver & Roeland, 2010).

The global meaning system of reformed pietists is characterized by a large focus on God’s providence (Hijweege, 2004). Furthermore, they regard the Bible as God’s word from cover to cover and strongly focus on Reformed doctrine and sanctification in all domains of life (Brienen et al., 1986). Humans’ nullity and guilt before God are contrasted to His omnipotence and righteousness (see, e.g. Schaap-Jonker et al., 2017). Only through a way of true faith, one can be reconciled with God and ‘taste the joy of His grace’. It can be expected that the COVID-19 pandemic will somehow be linked to the key notions of grief about sin, debt and guilt, in the light of God’s grace and forgiveness: *‘an emphasis on personal, self-examination, experience of faith: grief about sin, experience of guilt before God, inability to pay its debt, experiencing that one cannot hold their own before God, displeasing oneself, tasting the joy of God’s grace’* (van der Knijff, 2019, pp. 99–132).

Narratives about God and humankind reflect certain representations of God. Although God representations are affect-laden or experiential in quality (Zahl & Gibson, 2012) and thus are related to attachment and personality characteristics, personal reflections of narratives about God and humans are also shaped by doctrines or propositions within one’s church community, mediated by key figures such as ministers. In the individual experience, multiple God representations may exist simultaneously. Latent God representations can become more manifest due to changing contextual factors (Schaap-Jonker et al., 2017). This may imply that the pandemic affected the development of certain prominent representations of God and changed their functioning in the context of coping and meaning making.

People may uphold certain God representations as part of their religious coping — wherein for instance, also rituals, prayers and contemplation are implemented — but it will not always enhance their well-being. Certain representations may create resilience, but may also very well have paralyzing effects. In other words, religious coping methods can be *helpful or harmful* (Pargament et al., 2013). Positive religious coping which focusses on comfort, intimacy and closeness with God is often associated with fewer psychological distress (see Pargament et al., 1988; Phillips et al., 2004 in Pargament et al., 2013), while negative coping (such as beliefs related to God as punisher, or the devil being in control) is often related to higher levels of distress, as it, for instance, may lead believers to question God’s omnipotence (Pargament et al., 2011 in Park & Blake, 2020). However, this is not necessarily the case. There is empirical evidence that within reformed pietist circles, notions of sin and judgement do not always render psychological harm; God’s ruling and punishments may actually have supportive connotations (see Braam et al., 2008; Schaap-Jonker et al., 2017).

In a parallel study among key figures and ministers of the Dutch reformed pietist community (Cinjee & Schaap-Jonker, 2021), we found evidence that narratives about *God as ruler and punisher* were very much present during the pandemic. Also, the pandemic was interpreted as *a (wake-up) call to repentance* and/or a judgement for individual sinfulness or collective sins (Cinjee & Schaap-Jonker, 2021)**.** These notions do not always imply unbeneficial or harmful effects for the community members, as punishment, ordeal and tragedy can be seen as part of God’s benevolent plan (Pargament et al., 2013), and therefore have positive psychological effects if one believes one is safe in God’s hands (see also Park & Hale, 2014, p. 192). The crisis situation may, in this regard, show us whether coping has led to resilience. Besides God representations, other elements in the meaning making may prove to be very important for religious people to ‘hold on’, to process the ongoing situation, or to regain (mental) energy. Such sources of meaning and resilience are in need of further empirical examination.

### Aim and Research Question

In our current study, we aim to get insight into the perspectives of community members and address *(1)* how they dealt with encounters with COVID-19 and how they reflect on it *(2)* their interpretations of the religious meaning of the pandemic and what they believed God ‘has to say’ to them and/or to the world *(3)* what their main sources of resilience were and which role their Christian worldview and church community played in their perspectives. The main research question is as follows: *How did members of the reformed pietist community construct meaning regarding the COVID-19 pandemic, which role did their global meaning system play, and which elements in their meaning made were (main) sources of resilience?*

The aim of this qualitative study is not to give an overarching view of all narratives used by community members, but rather to trace how processes of meaning making are concretized in this specific situation. In the reformed pietist community, faith is (supposedly) present in all domains of life. Hence, we want to examine how the crisis has led reformed pietists to use typical narratives and interpretations, or maybe structure their own ones instead.

## Methods

### Interview Setup

We interviewed seven people from the Dutch reformed pietist community. We searched for respondents via networks of peers and colleagues as a starting point and provided them a general invitation letter about the purpose of the interview. These people could then also send this letter to their acquaintances (snowball sampling). Thirteen people, from various denominations and age groups, responded to the call. More men than women had responded to the letter, and in the end, a few of the female respondents dropped out. We selected five males and two females for the interviews; three were in their twenties, three were of middle age and one was in their seventies. None of them had former direct ties to the interviewer.

With this selection, we covered the spectrum of reformed pietist church denominations and selected respondents from the: Restored Reformed Churches (*n* = 1), Reformed Congregations (*n* = 2), Reformed Congregations in the Netherlands (*n* = 2), Old Reformed Churches (*n* = 1), Christian Reformed Churches (*n* = 1). Specific characteristics of the interviewed respondents are slightly altered in this paper, so that they are not retractable or recognizable, see Table 1.

**Table 1:** Characteristics of respondents (anonymized)

Resp. no	Gender	Church	Age	Abbreviation
1	Male	Restored Reformed	20	M20RRC
2	Female	Reformed Congregations	50	F50RC
3	Male	Reformed Congregations in the Netherlands	50	M50RCN
4	Male	Christian Reformed Churches	25	M25CRC
5	Male	Old Reformed Churches	70	M70ORC
6	Male	Reformed Congregations	25	M25RC
7	Female	Reformed Congregations in the Netherlands	60	F60RCN

Total N = 7

We used a semi-structured approach, whereby the interview was separated into three segments, (1) dealing with the crisis (roughly 15 min), (2) religious worldview, God and Bible, community life and narratives within the own community (roughly 20 min), (3) reaction to quotes by key ministers and figures of the community interpreting the crisis (roughly 20 min). We provided respondents an information letter which stated the goals of the research and how the data would be used and subsequently asked them to sign a declaration of consent. We hereby conformed to the university’s ethical guidelines. The interviews were held in the period January–April 2021, either at the respondent’s place of residence, or online via Zoom. Interviews were subsequently transcribed and coded in the months March and April. We transcribed mostly *clean verbatim*, so only when deemed relevant, respondents’ hesitations or long pauses were implemented in the transcription.

### Coding

For coding our data, we constructed seven main categories or ‘supercodes’ in NVivo (QSR, 2020), based on both the theoretical literature, as well as the insights of a parallel study (Cinjee & Schaap-Jonker, 2021). These supercodes are the following: (1)* dealing with suffering, *(2)* God representations, *(3.1)* meaning making: initial feelings, *(3.2)* meaning making: feelings in hindsight, *(4)* theological interpretations, *(5)* attitudes towards God, *(6)* church community and *(7)* themes.* Within these categories, we created ‘subcodes’ based on what the respondents brought up (inductive coding, bottom-up). Some of the subcodes appeared more often than others. For instance, under the supercode ‘God representations’, we added a few subcodes. The most prominent ones were: ‘God is above all things’ (11 references) and God ‘has a means with everything’ (8 references), see also Table 2. The full list of coding can be sent by the authors upon request. We evaluated the conversations with respondents through a lens of meaning construction: what was their primary source of meaning, did they use religious frames and religious rhetoric to interpret the crisis?

**Table 2:** Themes, interview segments, paragraphs and codes

Theme	Interview segment	Paragraph	Main supercodes and subcodes
Coping	Part 1	3.1	
		3.1 Coping with the disease: personal situations	(1) DEALING WITH SUFFERING
			(2) GOD REPRESENTATIONS
			*- God is above all things (11 refs)*
			*- God has a means with everything (8 refs)*
			(3) MEANING MAKING (1. Initial feelings)
			(7) THEMES
			*- repentance of sin (9 refs)*
			*- everything to God’s honour (5 refs)*
Meaning making	Part 2 & 3	3.2	
		3.2.1a The cause of the pandemic: concrete causes	(3) MEANING MAKING (2. Feelings in hindsight)
			(4) THEOLOGICAL INTERPRETATIONS
			*- eschatological (8 refs)*
			*- ecological (5 refs)*
		3.2.1b General causes (malleability)	(3) MEANING MAKING (2. Feelings in hindsight)
			(7) THEMES
			*- malleability (10 refs)*
		3.2.2. What it has to say to us: a call and a punishment	(4) THEOLOGICAL INTERPRETATIONS
			*- calling (7 refs)*
			*- call to repentance (7 refs)*
			*- punishment (4 refs)*
		3.2.3 How people respond	(5) ATTITUDES TOWARDS GOD
Resilience	Part 1, 2 & 3	3.3	
		3.3.1 Community	(6) CHURCH COMMUNITY
			*- together-feeling is important (17 refs)*
			*- missed going to church (8 refs)*
		3.3.2 Anger and scepticism towards government	(7) THEMES:
			*- sceptic towards government (9 refs)*
			*- angry towards government (4 refs)*
		3.3.3 The future of the church	(7) THEMES:
			*e.g. oppression of the church/moral decay (8 refs)*

We have structured the results section as follows: first, we focus on the personal copings of people with COVID-19. Those results are related to the first part of the interview (‘dealing with the crisis’). Second, we look into meaning making, regarding both the reasoning on the cause of the pandemic, as well its relational element: what the disease ‘has to say to us’ and how people ‘should respond’. Those results are related to the second and third part of the interview. Third, we look into sources of resilience. Those are sources which helped people to ‘hold on’, to process the ongoing situation, or to regain (mental) energy. Those results are related to the whole interview. In Table 2, we show how the results section relates to the supercodes (and main subcodes) in our data collection.

### Quotes

We assessed what respondents thought about themes which were prominently mentioned by key figures and ministers from the community. We asked the respondents to react to a few quotes, written by those key figures and ministers, which were selected based on the data collection of a parallel study (Cinjee & Schaap-Jonker, 2021). The six quotes were presented in a PowerPoint (if the interview was held via Zoom) or read out loud by the interviewer (if the interview was held on location). They were separated in three sets of two, with an overarching theme. Their purpose was to evoke ideas and responses by the respondents. The interviewer explicitly stated that the respondent should not feel onerous about ‘judging’ quotes of ministers or key figures from their community, as the quotes were purely meant to trigger reactions on the topic. The names of the authors of the quotes were not mentioned. The selection of authors covers the whole range of denominations within the reformed pietist community. In the results section, responses to the quotes will not be separately evaluated, but integrated into the text.

#### God’s Calling

1a “If we regard this virus as a grave and insinuating call by God, then it is important to know what He has to say to us. Can we give an answer to that question? That is, first and foremost, a personal thing. We cannot answer this question for church and country, if we have not contemplated it ourselves: ‘Hear, O my people, and I will speak’ [Ps 50:7 NKJV].^2^“

1b “When the Lord visits someone, also with a disease, then that is a calling: we are not here [on this earth] forever. The Lord does not afflict wholeheartedly, but merely so that one would seek Him and serve Him. The question is then: do we, within the Church, also want to return to the “old normal” and do we ignore God’s calling by merely trusting a vaccine?”.^3^

#### Humility and Prayer

2a “Let’s pray that people will seek this God or return to Him. Unfortunately, you do not see that happening in the Netherlands yet. It seems as if our people have become insensitive concerning [the ways of] God”.^4^

2b “Therefore, do not pray the judgments away, but beg [Him] whether everything that happens to us, in our personal lives and in the nation, may lead to our sincere, personal repentance and repentance of our family members, but also of our land and people; that there may be a return to the God of our ancestors”.^5^

#### Hope and Expectation

3a “God’s creation is in labour pain. Through crises in the midst of this suffering creation we can see the birth contractions of the Kingdom. In this way, we have heard the footsteps of Christ approaching for centuries.”^6^

3b “The only true medicine in this sick world, especially in times of crisis, is the Christian hope: the perspective that believers have of the glorious future of the Kingdom of God. A hope that, on the one hand, focusses on a Creation that once will be renewed, but which, on the other hand, also places the current reality in a different light.”^7^

## Results

### Coping with the Disease: Personal Situations

The first response and initial reflection on the coronavirus occurred in March 2020. While the severity of the crisis was not yet acknowledged by the general public, one respondent had seen the first wave occur in her close proximity. *“It felt like the angel of destruction went through the streets… That’s how it felt.” (F50RC).* The respondents (five of seven) who lost a dear family member, had one of their family members on a deathbed for a while, or were themselves struck by COVID-19, all endured a lot of uncertainty and stress in those early times. They talked about distressing conditions in which, first and foremost, there was need for a practical response: they simply had to deal with what was going on. It was a time of uncertainty and prayer, but not a time of rigorous theological reflection. In hindsight, two respondents said that in those initial times, trust in God was lacking to some degree:

In that moment it was really… a time of prayer. What else could I have done? I do not recollect that I have drastically trusted in God’s providence. If I would, I would not have been so scared. I maybe should have… had more trust in God (M20RRC).

The questions regarding God and the ‘why’ of their personal encounters with COVID-19 illness or death often arose in a later stage for the respondents (a few weeks or months after an infection) and were mentioned: *“why did this happen to me?”, “why us?”*. The God representations *‘God rules’* and *‘God has an intention with everything’* were most prominent (for five respondents). A strategy of putting the personal experiences of suffering into a broader perspective provided a way out of the why-questions: *“My suffering is part of a larger plan of God… Everything is to His honour, we must accept our fate” (M25CRC).* By extending the individual God–human relationship, questions about personal suffering are put in a frame on a higher-than-individual level, wherein one neither could nor should understand the ways of God. This started by acceptance of ‘not-understanding’ God:

and I won’t have to try hard to understand it all, because, if I would understand Him, He would not be worthy to be worshipped’ (M25CRC), “I often think: I do believe that there is a God behind this, but whether we can also understand what God does… Then He would be comprehensible in our minds, then He is not God-worthy, then He is just like us… …Apparently there must be reasons for God to do this, that we ourselves don’t understand, but somehow make sense… for us… or for the world” (M25RC), “If it was/is to the honour of God, and if he [family member on a deathbed] even were to die, what shall we say? He [God] has a purpose with everything (F60RCN).

By extension, the far more rigorous step should be taken to also actively accept and ‘be thankful for’ the ways of God…* “Lord, here is my life. If you have work for me today, please grant it me. If you have no work for me, you could let me die.” (M25CRC). *The way of true ‘conversion’ is, however, essential. That is: to recognize that we are under the curse of God, also during the pandemic, and that His judgements are righteous. *“It may not be in our nature [to recognize that God decides over our lives], but if you do not agree with something [a judgment of God], make yourself agree.” (M70ORC)*. By acknowledging God’s righteousness, and not trying to comprehend why He works in certain ways (if you would understand Him, He would not be worthy of worship), the need for further reasoning is effectively tackled. The ‘surrender to God’ can exist in various gradations, from a mere acceptance or feeling of Divine guidance, to an active agreement or even gratification in His judgements. In the meaning making concerning personal experiences with COVID-19, such perspectives appeared to be quite prominent.

### Why this Pandemic? Reasoning on the Global Level

#### The Cause of the Pandemic

**a. Concrete Causes**

To examine how meaning was ascribed to the pandemic, we let respondents react to two quotes (3a&3b) that were related to plausible interpretative frames on a global level, namely *(1)* an eschatological interpretation (COVID-19 is a sign of the end of times) and *(2)* an ecological interpretation. (COVID-19 determines humans at suffering of Creation due to their wrongdoing.) Following the reading of the quotes, respondents referred to the eschatological perspective, but mostly in a very general sense (six files) rather than concretely (one file). The pandemic was seen as ‘one of the many judgments’ and for sure not the *last* warning before the Final Judgement. Regarding the ecological interpretation, it was stated by a respondent that ecology should not be mixed too rigorously with the concepts of God’s omnipotence and guidance. Furthermore, he explained:

Creation being in labour pain is a Biblical notion I think, and that is correct. I do have problems with this quote [3a], as you also hear in certain circles that nature ‘revenges’ for the exhaustion of the earth. And, I can not see it entirely separate from this quote to be honest… …That is not something of 10 or 20 years ago… From the creation of the world onwards, there have been disasters and crises. In that sense this crisis brings us a step further to the second coming of Christ. But it surely is not ‘more’ than all of those other disasters that occurred… (M50RCN).

The overall interpretation of the pandemic becomes rather nuanced and generalized when one steps away from a concrete ecological or eschatological interpretation. COVID-19 then seems to be one of the many disasters that have occurred from the creation of the world onwards and is in that sense a step further to Christ’s second coming. The pandemic can, in such an interpretation, still be linked to the exhaustion of natural resources, as mentioned by another respondent, but in a more general way:

Yes… I myself think that it is a sort of… I read that corona is caused by a certain way of dealing with the earth, that the ecosystem is messed up and that that is its origin. A sort of scientific explanation… In a general sense I think – not specifically concerning the coronavirus – that Creation is in labour pain, yes. Natural resources are being exploited. The world cracks at her seams in my opinion, yes. (M25RC)

**b. General causes (malleability)**

Whereas very concrete eschatological or ecological interpretations were lacking, there appeared to be a more abstract and general cause of the pandemic, namely ‘malleability’. This theme was most prominent in the data, having been mentioned by six of the seven respondents. Most respondents regarded the pandemic either/or as an *(1)* event which affected the human illusion that we are strong and powerful beings, i.e. that we are alike gods, *(2)* event which triggered a sense of malleability — we humans, especially those in government positions, should be able to control this virus spread — and thus a lack of dependence on God. *“And I also believe that you live in a soap bubble, that you think everything will keep going fine… And that it [the soap bubble] will be poked through one day” (F60RCN)*. The government slogan *‘Together we will get corona under control’* was heavily criticized *(M70ORC, F50RC).* Regarding the cause of the pandemic, it could be said: humans (or specifically the people in the own church community) have been too ‘used’ to the situation as it was, they have enjoyed their luxury lives without too many worries. But now, this pandemic sets them still and determines them at their vanity. Also, in the overall response to the pandemic, as well as in the government response and their portrayal of the vaccine as the ‘light at the end of the tunnel’, we may have seen signs of humans thinking too highly of themselves.

If there is one thing which has become clear, then it is how unmalleable it [the response to the pandemic] is! And yet, everyone pretends – or at least a large part of our nation/in the church – that it is in fact the case. (M20RRC).

Only if we have truly received God’s grace in our lives, we can tackle the tendency to just progress and go forward: *“We are hardened… If God is not involved… we just want to go on again…” (M70ORC).* The concept of ‘malleability’ captures the essence of ‘what the pandemic has to say to us’, namely that people do not have everything in their own hands, but must surrender to God’s will.

### What it has to Say to us: A Call and a Punishment

Generally, the pandemic was seen as one of the judgements of God for human sinfulness, and a call to repentance. Just like previous pandemics were a call of God (such as the Spanish flu), so is COVID-19. The ‘calling’ was, by most respondents, seen as a personal thing: *“in the end, it is always the question: what does the judgment has to say to us personally” (M50RCN), “The calling is very personal, you may get sick of corona, but you may also die…” (F50RC)*.” The calling is very personal, as is every call to repentance by God.^8^ The pandemic should lead to repentance, be it repentance for the first time (being born again) or the continuous repentance of sins which is also needed for the true believers.

The extent to which this call or punishment should lead to very concrete changes in life is often not made clear. The calling is ‘one of the callings of God’, *“one of the low hanging judgments”* (*M70ORC)*, and must lead to a change in human attitude. But in establishing such a change, specific applications to the dynamics of the pandemic (and what God may have told people by means of its occurrence) are lacking. The reason for this was addressed by a respondent:

The necessity of true conversion… is despite the events in the world… the most important thing… …Everything which happens in the world, and in your personal life, and on the world stage: you may know that it is being guided. And the Lord has a meaning with everything, also with the coronavirus. (M50RCN).

This lack of a specific application can also go quite far, as the message of repentance is rarely linked to actuality at all:

Sermons in my church were like they were before (for the largest part), with the necessity to convert and being safe in Christ as a central message. Sometimes, yes, there are references to actuality. But this message transcends all actuality (M50RCN).

But if a message of repentance *transcends* actuality, will there ever be a situation in which a clear link is made between a specific calling or punishment by God and actual global events? *“The choice of Biblical texts has never depended on the coronacrisis. So… I actually find that a pity” (F50RC)*. And *if* it is called a judgement, why is the ‘call of God’ not being made more explicit? *“The judgments that occur, I see them as a call of God… But personally, if I were a minister, I would say: repent to God, go back to Him. Saying it somewhat more firmly.” (M25RC).*

In conclusion, we can state that notions of ‘calling, judgment and ordeal’ are very prominent, but there is little concrete application to the specifics of the pandemic. The pandemic is, on the one hand, seen as a special calling of God. On the other hand, the general call to repent of sin and flee to God is unaffected by tendencies in actuality.

#### How People Respond

Within the interpretative frame of reformed pietists, the pandemic is a call of God, who shows humans how insignificant they are and how they cannot manufacture everything in life. But to where should this call lead? As we saw in the results regarding the theme of ‘malleability’, people have the (sinful) tendency to ignore the calling of God. *“Everyone wants to get over it. We want to have the ‘old days’ back, and continue [life] in our own way” (F60RCN)*. The repentance to which is called stays on the ‘level’ of religious conversion of the individual; it does not easily translate into possible changes on aggregate/higher-than-individual levels (such as church, nation and worldwide). This may also have to do with less individuals who have turned towards God in the midst of this crisis, compared to others.

It [the crisis] leads to hardening instead of humility/humiliation’ (M20RRC, M25RC, F50RC). ‘It can lead to both conversion/repentance as well as hardening and now the tendency is towards hardening. After World War II the churches were filled with people, now it [this crisis] leads to decay. (M25RC, F50RC).

As COVID-19 has struck people everywhere in the world, it may still lead to changes in human attitude, which extend the reformed pietist group. The moral lessons that this crisis has taught regarding malleability have to do with global cross-cultural patterns of materialism and luxury, which are applicable for people in church (such as ministers, *as mentioned by F60RCN*), but also for each individual: ‘*how do I lead my life? What are my priorities? Am I prepared to discard wealth?’* (Matthew 19:21) (*as mentioned by F60RCN*). The strong focus on individual salvation in sermons may stand in the way of such broader perspectives *(mentioned by F50RC, M25CRC):*

Look, the focus in preaching is purely on personal salvation… Then there is few space for [talk about] the practical things… That is what you miss of course…”,“I think that if you only preach about personal salvation, it can become very egocentric… While we should also think: What does God require from our lives, where does He have the right to?... …you will think in all your choices and daily business: is this what God wants from me in my life? (F50RC).

### Sources of Resilience

#### Community

The first lockdown had a drastic impact on the religious life of community members: churches were closed or held services with less than 30 people. People could listen to services from inside their homes. The importance of church life was stressed often (by six respondents), as sometimes it was stated that the church is ‘the place where the Lord works’. Now, the pandemic may have led some to ascribe more value to the community than they did before (at least consciously). The global meaning system of reformed pietists includes the notion that God can work in any context, and that He is not bound by place, time, or size of gathering. Respondents, however, seemed to gain a strong realization how important the community life actually had been for them, when they had to suddenly miss it or experience it in a reduced form due to COVID-19 restrictions. One of the respondents labelled his church community as a ‘community of saints’. When church services were getting a different shape and form, he found a different community through a group of colleagues with whom he discussed religious topics: *“Then I thought: well, this apparently is my new community of saints. I can express my Christianity there” (M20RRC)*. The ‘together-feeling’ appeared to be extremely important:

I found it [following services all by myself] becoming harder and harder, so the past couple of Sundays I regularly went to friends… a half or whole Sunday to attend the services, and I love it that you… have the together-feeling (M25CRC).

The importance of ‘letting the church services go on’ as normal as usual also had to do with this ‘undefinable’ feeling: *“To gather together to some place is far more a church-feeling than being at home” (M20RRC).* The act of going-to-church was also a ritualistic event, which provided structure in the week.

For me, the community-feeling has an added value, also concerning the church services. It is also a bit of rhythm, in the beginning [of the first lockdown] I handled it too easily, I got out of bed, put on my sweatpants and sweater, sat down and on exactly 10 o’clock I was ready, with a cup of coffee next to me. So in that case this community-feeling is lacking, and it is also the case that you have no regular preparation for your service. Taking a shower, putting your costume on, that already helps. So that is what I have been doing recently. Then you take a more ‘conscious’ approach. (M25CRC).

This ritualistic event seemed to comprise more than just a matter of ‘the outside’ or entourage. The respondent labelled his dealings with the church service as a *‘more conscious approach’*. Arguably, such an approach can be necessary for some community members to experience a true connection to God’s presence and holiness. The pandemic has reduced the religious experience to its mere core, but has also shown which elements are quite essential for its effectiveness.

#### Anger and Scepticism Towards the Government

Respondents furthermore tended to mention their views on government policy. In the data, we saw a distinction between perspectives on the crisis during the first lockdown vis-à-vis the second lockdown. Whereas in the first lockdown, perspectives on the government regulations mostly fell in the classic frame of ‘obeying the government as a servant of God’ *(“God has placed the sword in the hands of the government… …to protect the good”* (Art. 36 Belgic Confession)), this became different since the second lockdown; all respondents were rather sceptic about elements of the government’s approach.

The government may be seen as God’s servant, until their policies go against God’s Word. But when is that point being reached? (see Romans 13: 3–4). If one believes this point is reached, anger towards the government may act as a source of resilience, as a failing government could be contrasted to an all-good God.

In Romans 13 it is written that the government is there for our good, and I really do not have the impression that prohibiting people to enter the streets [the curfew], and visiting other people, is ‘the good thing’ at this moment… …People nowadays are very lonely, ‘together’ we should solve this [the pandemic], but people have not been as lonely as they are today”. That is my vision: I believe that at some point the limit is reached. It is no longer for our good, so I do not know to what extent a government is still a good government”……” I have a general feeling of calm… The lockdowns are horrible though… So in some sense there is also restlessness… But it really is more anger towards the government that I feel inside me than callings for God’s help, as I know God is good. (M20RRC).

Now, the strong contrast of a failing government to an all-good God may lead members to put more trust in God’s hands, but it also legitimizes negative sentiment to those in government positions. Certain perspectives on the government, but also on vaccinations, for instance, may act as sources of resilience which actually transcend the individual level and be more accurately placed on the level of the community. That resilience and fear are group processes of a marginalized community in a secular context, came more explicitly to the surface regarding perspectives on ‘the future of the church’ (3.3.3).

#### The Future of the Church

When respondents were asked about the origins of the pandemic, they often subtly mentioned certain tendencies in the church, the nation and the world, which they found very worrisome. Behind their words seemed to hide fear for the position of Christians in the Netherlands, as they are further shifting to a marginalized or pressured position in society. We found four different cases (quotes from respondents) and will briefly evaluate resembling patterns and overlap.

CASE 1: “There are things happening of which you really say: the world is going further away from God, I mean: it is still acceptable… to mention the freedom on the schools about the opinion on homosexuality, and then they say ‘you can no longer reject’ [teachers based on being engaged in a same-sex relationship], if you apply to a reformed pietist school… Those are all things of which I think: what is happening? Everything has to continue… Christianity is being pushed to the sides more and more…” (M70ORC)

CASE 2: “**Are you sceptical regarding the future?”** Yes, pretty much. You could say, yes it is doomsday thinking… You know, there is a God above all things… But on the other hand: you see with the gender-debate, and how people are abandoning Christian beliefs, I think that is the biggest worry…” (F60RCN),

CASE 3: “Yes, I myself was not thinking about ‘what we do wrong as a nation’, I more so think that: indeed sins are occurring… that is what I think, and I see that it is, in my opinion, secularizing more and more, now also with the fuss about gay conversion practices, the identity of Christian schools, you learn about it more and more in the news… …So I do think that in that sense there is a sort of hardening, yes.” (M25RC)

CASE 4: “The only thing that I can say is – but yes, that is not something of merely the past year, it has been playing a role since long ago – that there is so much in society and our legislation that goes against the word of the Lord, think of abortion, certain legislation regarding marriage…” (M50RCN)

The four cases hold a strong resemblance: there is a focus on ethical issues, legislation and general tendencies in society which ‘go against the word of the Lord’; there are concerns about the position of Christians and Christianity. Now, the mentioned worries are all on the societal level. Respondents were worried about Christianity in the Netherlands as such, and general shifts in thinking about gender, sexuality and moral views. The uniformity in these concerns may, arguably, lead to a stronger sense of community for reformed pietists in the near future, further contrasting the Christian minority to secular society. In this process, there may be less focus on internal divisions and more on external threats. This could be a source of resilience too — and thus be helpful — but may also lead to a lot of negative out-group sentiment — and render harmful effects.

## Discussion

In this study, we explored the processes of meaning construction for seven Dutch reformed pietists in the context of the COVID-19 pandemic.

First, we explored how people coped with the disease of COVID-19 when they encountered it and which narratives about God they upheld in the aftermath of such an encounter. These results are a strong indicator of situational meaning, as the respondents described their initial feelings, experiences and reflections of a situation they had never faced before. In dealing with encounters of COVID-19, the main theological interpretation was determined by the (formal) ‘knowledge’ that there is a God who steers all things, and who has a means with all that occurs in the world. Referrals to ‘God being above all things’ and ‘nothing going beyond the boundaries of His providence’ detach from too specific questions about His dealing with our current world in the midst of the pandemic. For every individual, a pressing issue which ‘transcends actuality’, however, was the need for conversion/the urge to repent. Within reformed pietists’ global meaning system, there is a strong focus on the broken God–human relationship (due to human sinfulness), so that one can say: ‘we are all sinners, and thus deserve God’s punishment’. The fact that God has a ‘goal’ with everything means that He has something to say to someone when the pandemic hits them in their personal lives. This, however, does not provide an answer to why the pandemic occurred in the first place.

To go more into detail about the nature of the pandemic, respondents were asked how they interpreted the pandemic ‘as a whole’. Respondents shared their conscious reflections about the cause of the pandemic, what they thought it had to say to them and how people (should) respond. Here, elements from the global meaning system (general narratives from the community about God and humans, judgement and repentance) were applied to the pandemic. Ecological and eschatological interpretations could be applied on a global level, the same level on which the pandemic occurs, but were rarely used. There are many plausible reasons why this is the case. In the opinion of some reformed pietists, eschatological interpretations may have strong connotations with complot theories, evangelicalism, or may themselves go against God’s own word (as Christ will return as a thief at night, Revelations 3:3). Ecological interpretations, on the other hand, may have connotations with the narrative of climate change, and fundamentalist protestants are known to be less likely to believe the conclusiveness of climate science compared to non-religious people (Evans & Feng, 2013). In contrast, an interpretative frame that was used by our respondents was the theme of ‘malleability’. This theme is universal and can be applied to the own context. (We think we have everything in our own hands, see situation X in my church, or the behaviour of person Y.) It can be linked to both the origins of the pandemic, as to its response, but can also be used as a starting point to discuss worries about the future of the church and the nation.

We witnessed two essential ‘clashes’, wherein the global meaning system of reformed pietists seemed to connect general notions of calling and repentance to the worldwide pandemic. First, the idea that a message of repentance may transcend actuality, instead of being necessarily changed, specified or intensified by it. Second, the realization that the focus in reformed pietist preaching is merely on the individual God–human relationship and seems to lack an explanatory frame which goes beyond this level. The pandemic is a worldwide phenomenon, and within reformed pietists’ global meaning systems, disease and disaster may be linked to sins of the collective. At the same time, notions of kneeling before God’s presence and repentance were only made explicit on the individual level (as all individuals need to repent), but were not made explicit on the global level (ecological, eschatological). On the short term, this leads to resilience and useful interpretations of the pandemic. However, on the long term, the incongruence creates ambiguities, which undermine people’s efforts to make sense of how to concretely organize their lives and adapt their worldview in a post-pandemic era.

Results suggest that the most important elements in the situational meaning were *(1)* to rest in the Lord, to accept that He is above all things and has something to say to us with this pandemic *(2)* to deal with suffering and to maintain busy in regular daily life *(3)* to create own forms of rhythm/regularity, ritual and to continue community engagement. We too witnessed that both existential worries and resilience can exceed the individual level, namely the community (reformed pietists vs. secular) level, as there is a broad-based fear about secularizing trends. Respondents mentioned moral decay, and oppression of Christians and the Church as main sources of concern.

### Limitations

Our study had a few limitations. We interviewed more male than female respondents, as far more men reacted to the invitation, and a few of the female respondents dropped out. The fact that it was mostly men who responded may have to do with the explicit theological interpretations which were part of the questionnaire, which were also addressed in the initial information letter. It may be the case that reformed pietist men are more willing to expand on this, which would fit in with differences in gender roles within this community. However, in our small selection of interviews, we could not identify specific gender differences in perspectives on COVID-19.

Another limitation of the study was its retrospective design; we asked respondents to look back one year in the past, while their perspectives could surely have changed in the meantime and affected the way they reflected.

## Conclusion

This study has set an important first step in establishing how narratives and meaning making in the Dutch reformed pietist community were not only propagated top-down (via ministers and key figures), but were also shaped bottom-up, considering the experiences people themselves had with COVID-19. A further examination of the interaction between individual and societal processes of meaning making, such as secular interpretative modes, is needed (see also Park & Blake, 2020, p. 21). Whether and how meaning and resilience transcend the individual level and are concretized on the level of the community deserves attention too. This requires an approach in which sociological trends of reformed pietists are further dissected and examined.

## Data Availability

The information letter and invitation to respondents can be sent upon request. The transcribed interviews can be shared only in so far as the respondent’s confidentiality can be ensured.

## Footnotes

1 We merely focus on the denominations/wings within denominations which can be also defined as ‘reformed pietists’ or ‘strict Calvinists’; we do not include the middle-orthodox or modern reformed churches. What we define as ‘reformed pietist’ are in Dutch often referred to as ‘*bevindelijk gereformeerden’* i.e. those churches and wings within churches which focus more on the personal ‘appropriation of salvation’ and ‘seperating preaching’ (*the dividing line between the redeemed and unredeemed goes through the church community too*). Those communities range denomination-wise from a relative orthodox wing within the Christian Reformed Churches and Restored Reformed Church to the conservative (Old) Reformed Congregations in the Netherlands.

2 SERMON2.1 (sermons and news articles are coded by the authors).

3 SERMON1.2.

4 RD4.

5 SERMON1.2.

6 RD7.

7 RD15.

8 One could even make a distinction between ‘chastening’ and ‘punishing’, depending on who is being affected*: “The Lord chastens His people and punishes those who are not His people…”(M50RCN)*.

## Code Availability

The coding schemes that were used for analysis can be sent by the researchers upon request.
